# Tissue adhesives for bacterial inhibition in extracorporeal membrane oxygenation cannulae

**DOI:** 10.1186/s40635-021-00388-6

**Published:** 2021-05-10

**Authors:** India Pearse, Amanda Corley, Yue Qu, John Fraser

**Affiliations:** 1 Critical Care Research Group, The Prince Charles Hospital and University of Queensland, Chermside, QLD Australia; 2 School of Nursing and Midwifery, Griffith University, Queensland, Australia; 3 Biomedicine Discovery Institute, Department of Microbiology, School of Medicine, Nursing and Health Sciences, Monash University, Melbourne, VIC Australia; 4 Department of Infectious Diseases, The Alfred Hospital and Central Clinical School,, Monash University, Melbourne, VIC Australia; 5 Adult Intensive Care Services, The Prince Charles Hospital, Chermside, QLD Australia

**Keywords:** Extracorporeal membrane oxygenation, Infection, Cyanoacrylate, Tissue adhesive, Bacteria

## Abstract

**Background:**

One of the most serious complications of extracorporeal membrane oxygenation (ECMO) therapy is ECMO cannulae infection, which can occur at quadruple the rate of central venous catheter infections, and significantly impact morbidity and paediatric mortality. The objective of this in vitro observational study was to assess antimicrobial properties of two *n*-butyl-2-octyl cyanoacrylate tissue adhesive (TA) formulations for bacterial inhibition at peripheral ECMO cannulae insertion sites.

**Methods:**

Antimicrobial properties were assessed using modified agar disk-diffusion (*n* = 3) and simulated agar cannulation insertion site (*n* = 20) models. Both assays used *Staphylococcus epidermidis* which was seeded at the edge of the TA or dressing. Microorganism inhibition was visually inspected and evidenced by the presence or absence of a TA bacterial inhibition zone at 24 and 72 h.

**Results:**

Both TAs provided effective barriers to bacterial migration under cannula dressings, to cannula insertion sites and down cannula tunnels. Additionally, both TAs demonstrated distinct zones of inhibition produced when left to polymerise onto agar plates seeded with *S. epidermidis*.

**Conclusions:**

*N*-Butyl-2-octyl cyanoacrylate TA appears to inhibit bacterial growth and migration of *S. epidermidis*. Application of TA to cannulae insertion sites may therefore be a potential bedside strategy for infection prevention in ECMO cannulae, but requires further testing before being used clinically for this purpose.

## Introduction

Extracorporeal membrane oxygenation (ECMO) is a form of mechanical circulatory support which provides cardio-respiratory assistance to critically ill patients refractory to conventional treatment therapies [1]. ECMO therapy is delivered via large-bore cannulae which are inserted into the patient’s great blood vessels and must remain in situ, sometimes for prolonged periods, until the patient no longer requires ECMO support. Diligent management of these cannulae, including effective antisepsis, is imperative to reduce the risk of cannula-related infections, including local and cannulae-related bloodstream infections, which negatively impact patient outcomes [2].

Infection is one of the most serious complications of ECMO [3], with up to 64% of patients receiving ECMO developing nosocomial infection during their treatment [4]. Such infections are often associated with longer periods of mechanical ventilation and ECMO support [4, 5], leading to longer hospital stay [4, 6] and increased mortality in the paediatric cohort [7]. Patients undergoing ECMO are at an increased risk of developing a nosocomial infection due to alterations in their immune function, use of broad spectrum antimicrobial agents, and presence of multiple portals of microorganism entry [8].

ECMO cannula-related infections are not commonly reported [9], most likely due to inherent difficulties in detecting and diagnosing such infection in patients receiving ECMO [10]. Contributing factors to these difficulties include the underlying disease process, systemic inflammatory responses to the presence of the extracorporeal circuit and the fact that cannulae cannot be easily replaced if infection is suspected [6, 11] as it carries high risk for the patient [11]. It is therefore difficult to definitively diagnose and treat suspected cannula-related infections [10, 11]. Nonetheless, ECMO cannula infections are estimated to occur at a rate quadruple that of other intravascular devices [9].

Cannula infections are caused by microorganisms proliferating on the inner and/or outer surface of the cannulae [12]. Many of these microorganisms have the ability to form biofilms, a microbial growth model highly resistant to conventional antibiotics [13, 14] and the patient’s innate immune defences [12, 14]. The natural lifecycle of biofilms, including that of *Staphylococcus epidermidis* which is one of the main organisms responsible for ECMO cannula infections [2, 4, 9], involves microorganisms detaching from the main biofilm as planktonic cells and reseeding different anatomical locations [14]. When this process occurs for biofilms on intravascular devices, including ECMO cannulae, patients may develop a potentially devastating bloodstream infection [14]. Current data suggests that approximately 12% of cannula infections result in bloodstream infections [3].

A portal of entry for microorganisms on to cannulae surfaces is through the cannula insertion site [11, 12] as a result of inadequate skin antisepsis [14]. As such, particular attention is paid in clinical practice to antisepsis of the cannula insertion site and maintenance of sterility of the site throughout the duration of ECMO treatment. Current practice is to use chlorhexidine-based preparations for insertion site antisepsis, and to cover the site with a sterile, transparent, semipermeable dressing [15] to promote site sterility and provide an element of cannula securement. However, there are no standardised, evidence-based guidelines specific to dressing and securement of ECMO cannulae, with recommended interventions only reflecting general infection prevention practices used in the critically ill [10]. As such, infection prevention practices vary across the world [15].

Evidence has recently emerged indicating that cyanoacrylate tissue adhesives (TAs) possess antimicrobial properties [16–18], and securement abilities which may reduce micro-motion and pistoning of intravascular devices by sealing the insertion site [19]. As such, TA may be a potentially useful method of reducing intravascular device infection [20], including those related to ECMO cannulae [21]. However, there is very limited evidence pertaining to TA for ECMO cannulae infection prevention, and additional, high-quality pre-clinical data need to be generated before TA may be considered for clinical management of ECMO cannulae. The aim of this study was to determine the antimicrobial properties of cyanoacrylate TA formulations when used at cannula insertion sites in an in vitro ECMO cannulation simulation.

## Methods

*N*-Butyl-2-octyl cyanoacrylate formulation TAs (Glubran® Tiss2, GEM, Italy and SecurePortIV™, Adhezion Biomedical, USA) were chosen for this study as this formulation has demonstrated superior securement properties (tensile strength and flexibility) compared to 2-octyl cyanoacrylate formulations in previous in vitro ECMO cannula securement testing conducted by our group. Antimicrobial testing of 2-octyl and *n*-butyl cyanoacrylates have previously been undertaken [16, 18].

### Antimicrobial susceptibility testing

A modified agar disk-diffusion-based antimicrobial susceptibility assay was performed to examine the potential antimicrobial activities of TAs, by following the Clinical and Laboratory Standard Institute (CLSI) guideline M02-A11 [22] with slight modification. *Staphylococcus epidermidis* ATCC 35984 (RP62A) was chosen for this study as this strain represents a species commonly associated with catheter-related bloodstream infections [20]. Inoculum of *S. epidermidis* was prepared by growing cells for 5–6 h to a log phase in nutrient broth. The suspensions were adjusted with 0.85% sodium chloride to final optical density readings of 0.1 with a spectrophotometer at 600NM. The adjusted suspensions were used to inoculate Muller–Hinton agar (MHA) plates. Twenty microliters of either Glubran^®^ Tiss2 or SecurePortIV™ TA was placed at the centre of two inoculated MHA plates, in addition to a control plate which was inoculated but did not have TA applied. The plates were incubated in a humid chamber for 24 h at 35 degrees Celsius and the inhibition zone of bacterial growth around the TA was imaged. Antimicrobial effects were identified through presence or absence of a microbial inhibition zone on the inoculated plates.

### In vitro simulation of ECMO cannulation

In vitro simulation methods were modelled on a previously conducted study by our group [21]. Dextrose tryptone purple bromocresol agar (tryptone 10 g/L, dextrose 5 g/L, bromocresol purple 0.04 g/L, and agar 15 g/L) was prepared and a 5 cm section of 23F Bio-Medicus femoral venous single-stage cannula (Medtronic Inc, Minneapolis) was placed in a petri dish (100 mm × 15 mm), with one end siting in the centre of the petri dish and the other end resting on the dish rim. Fifty millilitres of melted agar was cautiously cast into the dish to cover most, but not all of the cannula. After the agar solidified, the cannula was carefully and aseptically pulled out from and replaced into the agar to create a subcutaneous tunnel, mimicking the tunnel created by percutaneous insertion of an ECMO cannula, at an angle of approximately 12.5 degrees. The exit site of the cannula was secured with one of the following methods: (1) transparent dressing (Opsite™, Smith and Nephew, London, UK) only (standard care), (2) Glubran Tiss2 TA with transparent dressing, (3) Glubran Tiss2 TA without transparent dressing, (4) SecurePortIV TA with transparent dressing, and (5) SecurePortIV TA without transparent dressing. This was repeated four times for each dressing combination.

Two to three drops of TA were applied at the cannula entrance site and allowed to completely polymerise, before being covered by a dressing, or left uncovered depending on securement method being tested. Crinkles in the dressings which appeared on dressing application were tolerated to mimic the application of an ECMO dressing in standard clinical practice. A 10 μL aliquot of *S. epidermidis* RP62A at 1 × 10^8^ CFU/mL was then seeded at the outermost edge of the securement method (this included at the outermost dressing edge for standard care plates). The seeded plates were incubated in a humid chamber at 35 degrees Celsius for a total of 72 h. The plates were examined and photographed for bacterial growth and migration at 24, 48 and 72 h. Bacterial migration was indicated by the growth of bacterial colonies on the plate as imaged using a digital camera at 24 and 48-h time points, and with a Fuji LAS-3000 imaging system at the 72-h time point. Agar pH colour change from purple to yellow was also utilised as a secondary method to visualise colony growth.

### Statistical analysis

Sample size calculations were based on similar testing previously reported [21]. Antimicrobial properties of the TA were visually inspected and evidenced by the presence or absence of a bacterial inhibition zone surrounding the TA. Results are presented descriptively.

## Results

### Antimicrobial susceptibility testing

**Fig. 1:**
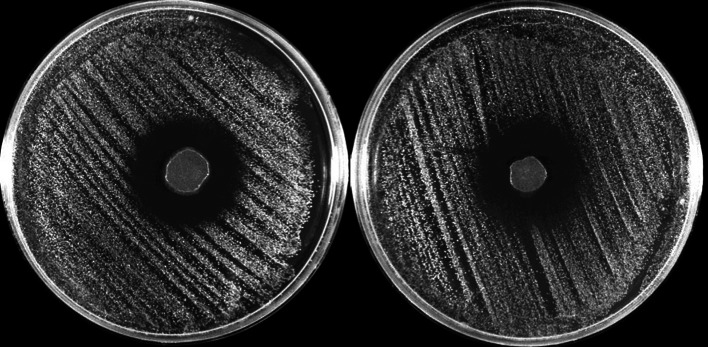
Antimicrobial effects of Glubran Tiss 2 and SecurePortIV on *S. epidermidis* after 24 h

An evident inhibition zone was found around both deposits of TA (Fig. 1) which was in contrast to control (see Fig. 1). There were similar antimicrobial effects observed for both TA formulations tested.

### In vitro simulation of ECMO cannulation

**Fig. 2:**
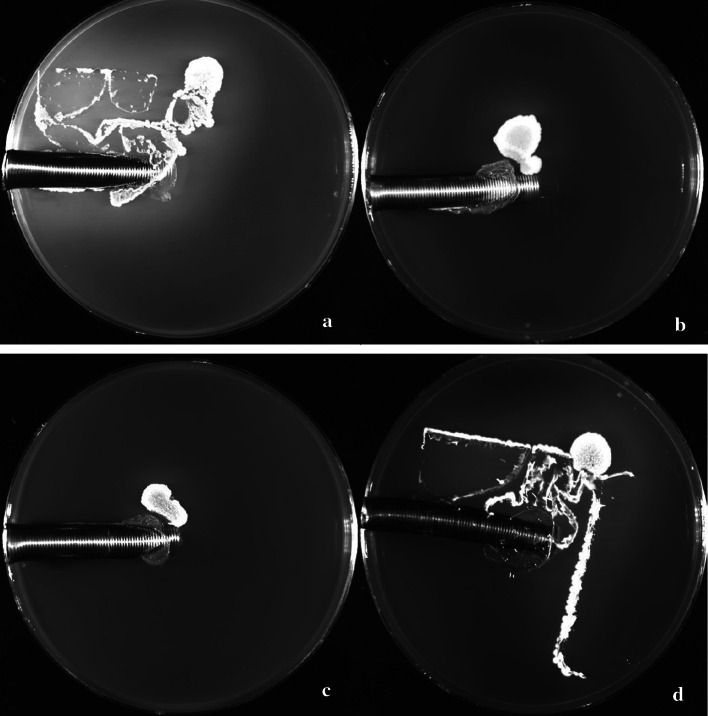
Bacterial migration according to securement method (**a** transparent dressing only, **b** Glubran Tiss2 only, **c** SecurePortIV only, **d** SecurePortIV and transparent dressing)

Cannula plates containing ECMO cannulae secured with TA showed complete bacterial inhibition both at the insertion site and down the cannula tunnel when challenged with *S. epidermidis* (Fig. 2b–d). There was no discernible difference, either visually or by agar pH colour change, in the inhibitory properties of the two TA formulations (Fig. 2b, c). In contrast, covering the insertion site with a transparent dressing without the use of TA facilitated migration of *S. epidermidis* from the seeding point along the fold tracks of the dressing to the insertion site of ECMO cannula within 24 h of seeding (Fig. 2a, d). Once past the exit site, the presence of the simulated subcutaneous cannula tunnel further allowed bacteria to migrate down the tunnel, reflecting the migration of bacteria in vivo (Fig. 2a). For the plates in which the ECMO cannula was secured with TA plus transparent dressing (Fig. 2d), bacterial migration underneath the dressing was evident, however the presence of the TA around the ECMO cannula insertion site effectively blocked bacterial access to the cannula insertion site and down the cannula tunnel.

## Discussion

This in vitro study demonstrated excellent antimicrobial properties of *n*-butyl-2-octyl cyanoacrylate tissue adhesive, using a modified CLSI assay and a newly developed ECMO cannulation simulation model. Both TAs demonstrated zones of inhibition noted on agar plates seeded with *S. epidermidis*, when compared with control. Additionally, both TAs tested provided an effective barrier to microorganism migration to the insertion site and down the tunnel of the ECMO cannulation simulation. This effect persisted with the addition of a transparent dressing over the TA, however bacterial growth was noted under the dressing. As such, our findings suggest that the application of *n*-butyl-2-octyl cyanoacrylate TA around peripherally inserted ECMO cannula as a strategy to reduce cannula-related infection warrants further investigation.

Our results add to the limited data currently published pertaining to TA use in ECMO cannula infection prevention and securement. In our initial in vitro study, we examined the use of first generation (*n*-butyl-2 cyanoacrylate) TA under similar test conditions [21], finding that TA possessed the same ability to inhibit bacterial migration to the cannula insertion sites and down the cannula tunnel. Since this study, new generation TA (*n*-butyl-2-octyl cyanoacrylate) formulations have been developed and are now widely used in clinical practice for wound closure and intravascular device securement. The present study aimed to determine whether this new formulation possesses the same antimicrobial properties as its predecessor.

Antimicrobial activity of *n*-butyl-2-octyl cyanoacrylate TA observed in this study aligns with other previous studies testing other cyanoacrylate formulations [16, 18]. The prominent exclusion zone noted around both polymerised TAs, as also seen in the studies by Wilkinson et al. and Prince et al. [16, 18], indicates a strong bio-incompatibility of *n*-butyl-2-octyl cyanoacrylate with *S. epidermidis*, even though this formulation has a slightly higher water content (0.2%) [23] than 2-octyl cyanoacrylate formulations (0.16%) previously tested [16]. This antimicrobial effect is postulated to occur through the diffusion of water from bacterial cells into the TA, leaving the bacteria deprived of water and subsequently unable to survive [16]. However, the exact antimicrobial mechanism of action is still unknown, and investigating this mechanism was outside the scope of this study.

Tissue adhesives have previously been used for securement and infection prevention in small-bore intravascular catheters [20, 24–26]. Tissue adhesives have also been shown to promote insertion site haemostasis in smaller intravascular devices [25]. Because of the securement, haemostatic and antimicrobial properties of TA, it may be a useful method of reducing overall failure of vascular access devices. The only sufficiently powered randomised controlled trial to test TA use in vascular access devices found no difference in peripheral intravenous catheter failure, due to infection or other causes, between TA and control [27]. There is currently, however, no clinical data specific to the use of TA for ECMO cannula securement, therefore adequately powered RCTs are needed to definitively determine TA’s efficacy in ECMO cannula infection prevention.

In addition to antimicrobial qualities, cyanoacrylate TA possesses high tensile strength, which facilitates a secure seal around cannula insertion sites [21]. However, *n*-butyl cyanoacrylates can be quite brittle [19]. As the addition of 2-octyl cyanoacrylate to *n*-butyl-2 cyanoacrylate TAs creates a longer alkyl chain and therefore increases the flexibility of TA bonds [19, 28], formulations such as *n*-butyl-2-octyl cyanoacrylate may more readily accommodate natural movement on human tissue while keeping the ECMO insertion site bacteria-free and the cannulas securely in place.

As stated previously, the secure and flexible seal provided by TAs is also reported to facilitate haemostasis at the insertion site of smaller intravascular devices with favourable results [25]. However, TAs have not previously been used in ECMO cannulae for haemostasis. There are concerns that, in patients who are actively bleeding from their cannulation sites (in particular, those patients who have undergone direct cut-down insertion), TA’s inhibition of active blood flow from vessels and out of the insertion site cause haematoma [29] and other associated complications. As such, TA application to cannulation sites may only be appropriate for those peripherally cannulated patients who undergo Seldinger insertion techniques which typically produce a tighter fit of the skin around the cannula and less bleeding [30].

There were several limitations to this study. Firstly, we only examined the antimicrobial effects (see Fig. 1) qualitatively and are therefore unable to quantitatively compare the difference between the two TAs. Additionally, a shortcoming of using CLSI-recommended zone of inhibition methodology to describe antimicrobial effects is that the activity against the microorganisms directly beneath the polymerised TA, if any, is not detectable [16]. We did not use an alternative method of quantifying the antimicrobial effects observed, therefore we cannot definitively comment on the antimicrobial effect underneath polymerised *n*-butyl-2-octyl cyanoacrylate in this study. Finally, only *S. epidermidis* was tested in this study as it is a microorganism commonly responsible for catheter-related bloodstream infection [20] and the results are therefore not generalisable to other microorganisms. It would be of interest to repeat this study using other species of microorganisms, such as other bacterial pathogens commonly seen in ECMO patients with bloodstream infection, including *Pseudomonas aeruginosa* and *Staphylococcus aureus*, and fungal pathogens such as *Candida albicans* to understand if the same inhibitory mechanisms apply.

## Conclusion

*N*-Butyl-2-octyl cyanoacrylate has strong antimicrobial properties against *S. epidermidis*, and therefore use of this formulation TA for bacterial inhibition at ECMO cannula insertion sites may be a promising method of infection prevention in patients susceptible to hospital acquired infections. However, the findings of this study require further investigation before they may be translated into clinical practice. Adequately powered randomised controlled trials are also needed to test the usefulness of TA use at peripheral ECMO cannula insertion sites as a strategy to reduce the incidence of cannula-related infection.

## Data Availability

The datasets generated and analysed during this study are available from the corresponding author on reasonable request.

## Abbreviations

ECMO: Extracorporeal membrane oxygenation
TA: Tissue adhesive
